# The Treatment of Impacted Biliary Concretions

**Published:** 1898-10

**Authors:** E. B. Jackson

**Affiliations:** Houston, Tex.


### THE TREATMENT OF IMPACTED BILIARY CONCRETIONS.

By E. B. JACKSON, M.D.,
HOUSTON, TEX.

This is quite a painful and serious condition. It is not possible to predict the number of days that an attack will last. The attack will often come on suddenly, though this is by no means always the case. If a stone of small size—say equal to a grape seed—leaves the gall-bladder in a quiescent state, so to speak, engaging the commune duct slowly, it may be two weeks in passing, and without producing great pain, since there may be ample space for the passage of bile through the duct by the angles of the calculus. And the stone may never be entirely impeded at any point in the duct, therefore the pain may be only slight. But if a large, round stone suddenly engages the duct and becomes at once completely impeded, we have pain the intensity of which I cannot with words describe. It is periodical—comes on every few minutes in the form of violent colic, but the cycles are often quite irregular. The patient may or may not have jaundice—much oftener not. There is, however, nearly always a certain grade of cachexia, due to chronic inactivity of the liver, and malarial toxemia. The face is usually muddy, the complexion spotted with patches of brown pigment. It is easy to see that such a stagnant condition of the system would readily aid the formation of gall-stones, namely, by favoring the exudation of mucus in the duct, by producing a catarrhal condition of the bile bladder itself, and by promoting the secretion from the liver of bile of such consistency as that its degeneration, when pent up in the gall-sac, most easily occurs, particularly insomuch as it must lie in contact with an unhealthy surface, and be shut off from the abdomen by blocking of the duct with mucus and abnormal cell proliferations due to catarrhal inflammation. If the gall-bladder be much enlarged it may be felt as an oblong tumor under the tip of the tenth rib. This being felt, it is easy to account for the tympanitis that is also seen in connection with a case. You must of course give morphine, for nothing else ever coming under our observation was sufficient to quell the insufferable pain; give it by all means hypodermically in one-fourth grain doses four times a day, and oftener if the case requires it. You must put on what we are in the habit of calling a turpentine stupe poultice. It is made of linseed meal wet with equal parts of ether and turpentine. You are to apply this over the gall-bladder. If you wish to shorten the passage of the stone by internal remedies, you must administer chionia for removing the *débris* from the duct and gall-bladder, and for normalizing the flow and consistency of bile. We believe also, from our experience with three recent cases, that it exerts a favorable influence in relaxing the grasp of the duct upon the stone, for the exit of each one of them seemed more readily accomplished than in many cases formerly treated when chionia was not administered, but the other measures herein recounted were applied. If you desire happy results from internal treatment, by all means prescribe one teaspoonful of chionia four times a day in hot water. There is but little doubt that this remedy, if used in advance of serious impaction, would, by its power in aiding the liver and bowels to resume their normal functions, prevent paroxysms of hepatic colic by preventing the formation of concretions in the gall-sac.

A word in reference to cholecystotomy, if the case becomes obstinate. If many ugly symptoms develop—such as approaching jaundice, extreme tenderness and tympanitis, extreme enlargement of the gall-bladder, and general tendency to prostration becomes imminent—it is best to operate and relieve the condition in this manner, namely: make a semi-circular incision large enough to get the hand in over the tip of the tenth rib, turning the flap upward, fixing it temporarily to the skin of the chest. Open the peritoneum and immediately pack it with flat sponges under the lower margin of the wound; now put a stout ligature through the most dependent margin of the liver, that it may thus be bridled upward by an assistant and maintained so throughout the operation; now make a longitudinal incision in the gall-sac, letting out all stones, mucus and disorganized bile; now remove the sponges—take care that all oozing is checked; then stitch the skin, the peritoneum and the margin of the incision in the gall-bladder all together, just as you would stitch a buttonhole were you a tailor; now insert a long rubber tube, long enough to reach from the wound to the patient's side-pocket, so that the drain from the gall-sac may be conveyed by the tube into a bottle that the patient can easily wear in his pocket all through the process of closure of the resultant fistula—sometimes requiring months. After this tube is inserted the wound can be closed around it and carefully packed with iodoform gauze and covered with oil-silk. The operation is simple and as easily and quickly performed as colotomy; and we have heretofore reported one successful case, and the patient is still living, this being the sixth year of comparative health which she has enjoyed.

